# Sclerosing Mesenteritis as an Uncommon Site of Involvement of IgG4-Related Disease: A Case Report With an Updated Review of the Literature

**DOI:** 10.7759/cureus.25041

**Published:** 2022-05-16

**Authors:** Michele Bertoni, Alessandra Giani, Silvia Tozzini, Massimo Edoardo Di Natale

**Affiliations:** 1 2nd Department of Internal Medicine, Ospedale Santo Stefano, Prato, ITA; 2 Pathological Anatomy Department, Ospedale Santi Cosma e Damiano, Pescia, ITA

**Keywords:** comprehensive diagnostic criteria, infiltration of igg4 plasma cells, elevated serum igg4, sclerosing mesenteritis, igg-4 related disease

## Abstract

Immunoglobulin G4-related disease (IgG4-RD) is an uncommon immune-mediated disorder most commonly involving the pancreas, lacrimal, and salivary glands. Immunoglobulin G4-related sclerosing mesenteritis (IgG4-RSM) is a rare site of involvement that usually mimics the imaging characteristics of mesenteric malignancies. Herein, we report a case of IgG4-RSM followed by an updated and comprehensive review of the literature. A 73-year-old woman presented with colicky abdominal pain in the right hypochondrium. The findings on contrast medium computed tomography (CMCT) showed a swelling of the mesenteric root with vascular structures surrounded by slightly contrast-impregnated tissue and irregular margins. The 18F-fluorodeoxyglucose positron emission tomography/computed tomography (18F-FDG-PET) showed an area of inhomogeneous and intense hypermetabolism of the mesenteric root. Hence, laparoscopic resection of the mesenteric root was performed to distinguish such masses from malignant tumors, obtaining specimens for histopathologic examination. The latter exhibited tissue infiltration with lymphocytes, IgG4-positive plasma cells, and fibrosis, indicating a diagnosis of IgG4-RSM in the presence of both elevated serum IgG4 levels and the aforementioned imaging findings. With steroid therapy, no clinical signs of re-exacerbation within a one-year follow-up were observed and serum IgG4 levels returned to normality. Aiming to evaluate the real frequency of IgG4-RSM in view of the 2017 Comprehensive Diagnostic Criteria (CDC) of IgG4-RD, we undertook a complete MEDLINE, EMBASE, Web of Science, and Scopus database search of all case reports of IgG4-RSM published so far. Such criteria were met in only six cases with a definite diagnosis. This case highlights the mesentery as a rare site of involvement of IgG-RD and allows us to advance knowledge of IgG4-RSM.

## Introduction

Immunoglobulin G4-related disease (IgG4-RD) is a systemic, immune-mediated fibro-inflammatory disease characterized by tissue destruction of multiple organs. It was recognized as a systemic condition for the first time in 2003 [1] and much still needs to be investigated about this entity. IgG4-RD is an uncommon disorder that can involve any organ of the body, such as the pancreas, lacrimal and salivary glands (mimicking Sjogren Syndrome), appendix, mesentery and lymph nodes, kidney, and prostate, and can cause retroperitoneal fibrosis, vasculitis, or coexist in sarcoidosis [2,3], as reported in two comprehensive reviews of the literature. It is noteworthy that the histopathologic features of this disease are very similar regardless of the organ involved [4]. With regard to immunoglobulin G4-related sclerosing mesenteritis (IgG4-RSM), it usually simulates the characteristic imaging of mesenteric malignancies and its preoperative diagnosis remains challenging. Herein, we report a case of IgG4-RSM followed by a thorough literature review on this condition. This article was previously presented as a meeting abstract at the 2021 National Congress of the Italian Federation of the Associations of Hospital Internists on October 3, 2021.

## Case presentation

**Figure 1:**
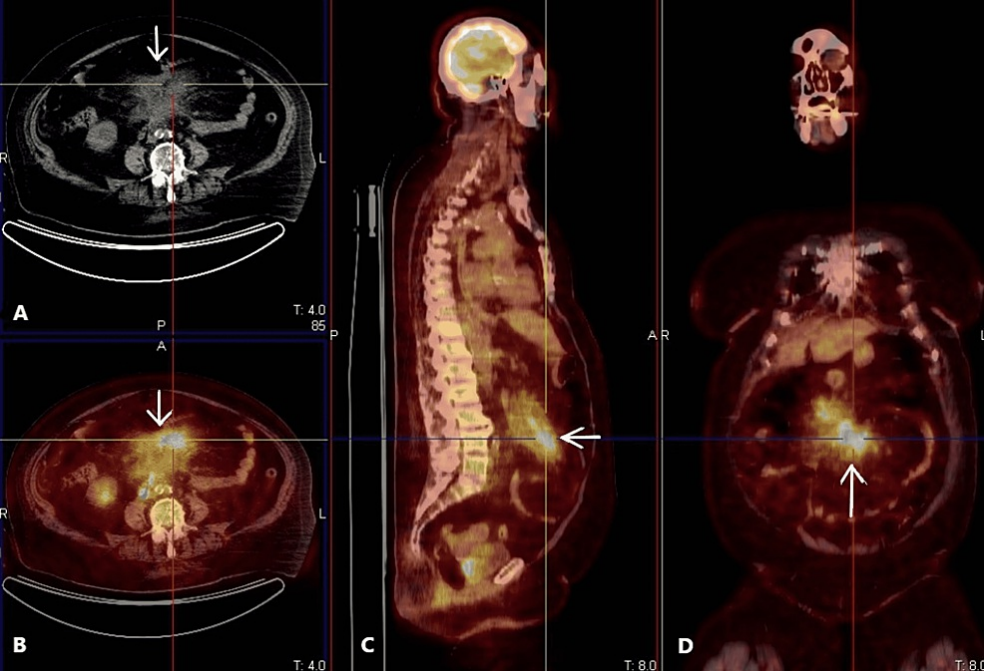
Pre-operatory imaging study of our patient with CMCT and total body 18F-FDG PET/CT. (A) Axial view of CMCT, contrast medium computed tomography, showing a swelling of the mesenteric root with slightly contrast-impregnated tissue and blurred and irregular margins (see arrow). (B) Axial view, (C) sagittal view, and (D) coronal view of total body 18F-FDG PET/CT, 18F-fluoro deoxy glucose positron tomography/computed tomography, showing an area of inhomogeneous and intense hypermetabolism of the mesentery root (see arrows).

The patient has given written permission for her details to be published. MA is a 73-year-old woman who underwent video laparoscopic cholecystectomy for gallbladder stones in May 2020. In the following two weeks, she began to experience recurrent episodes of abdominal colicky pain in the right hypochondrium, which subsequently radiated to the entire abdomen, along with nausea and vomiting. Her medical history was significant for an unprovoked pulmonary embolism for which she was treated with dabigatran 110 mg t.d. for six months one year ago. The patient denied fever, night sweats, and blood in the stool. Her physical examination was remarkable for abdominal distention with mild tenderness on deep palpation in the right quadrants with rebound tenderness. Hence, she promptly underwent an abdomen contrast medium computed tomography (CMCT), which showed a 9 cm × 8 cm swelling of the mesenteric root with vascular structures surrounded by slightly contrast-impregnated tissue and blurred and irregular margins with preservation of the fat around the mesenteric vessels and without bowel ileus or obstruction (Figure 1A). Ten days after, a total body 18F-fluorodeoxyglucose positron emission tomography/computed tomography (18F-FDG PET/CT) showed an area of inhomogeneous and intense hypermetabolism of the mesenteric root (Figure 1B, 1C, 1D).

The patient underwent a series of blood tests, the most significant of which are reported in Table 1.

**Table 1:** Most significant blood tests performed to the patient. CRP: C-reactive protein; ESR: erythrocyte sedimentation rate; IgG: immunoglobulin G; IgG4: immunoglobulin G4; CEA: carcinoembrionic antigen; Ca 19.9: carbohydrate antigen 19-9; Cyfra 21.1: cytokeratin 19 fragment; NSE: neuron-specific enolase.

Test	Results	Reference values
Hemoglobin	9.4 g/dL	12-16 g/dL
Sideremia	18 µg/dL	36-150 µg/dL
Ferritin	226.5 ng/mL	11-165 ng/mL
CRP	25.13 mg/dL	< 0.50 mg/dL
ESR	88 mm/hr	< 25 mm/hr
Fibrinogen	1,027 mg/dL	200-400 mg/dL
Gammaglobulinemia	1.3 g/dL	0.68-1.5 g/dL
Serum IgG	1,094 mg/dL	690-1,400 mg/dL
Serum IgG4	145 mg/dL	8-140 mg/dL
CEA	1.4 ng/mL	< 10 ng/mL
Ca 19.9	13 UI/mL	< 38 UI/mL
Cyfra 21.1	14 ng/mL	< 33 ng/mL
NSE	5.5 ng/mL	<15 ng/mL

They showed moderate inflammatory anemia, an increase in acute phase reactants, and an increase in serum IgG4 levels. The levels of the main tumor markers were normal. 

**Figure 2:**
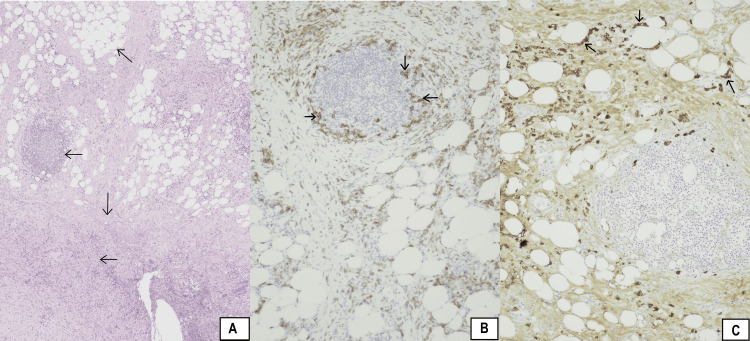
(A) Conspicuous chronic inflammatory infiltrate and collagen deposition in mesenteric adipose tissue (10×), (B) IgG immunostained section with many IgG positive plasma cells (40×), and (C) IgG4 immunostained section with many IgG4 positive plasma cells (40×). (A) Horizontal arrows indicate inflammatory lymphoplasmacytic infiltration. Vertical arrow indicates collagen deposition. Oblique arrow indicates mesenteric adipose tissue. (B) Arrows indicate IgG positive plasma cells. (C) Arrows indicate IgG4 positive plasma cells.

As it was difficult to distinguish the mass from abdominal malignant tumors such as malignant lymphoma, peritoneal carcinomatosis, metastatic carcinoid tumor, gastrointestinal stromal, carcinoid tumor, desmoid tumor, and metastatic adenocarcinoma, laparoscopic resection of the mesenteric root was performed in order to obtain specimens for the histopathologic examination. The histopathological examination exhibited a conspicuous chronic, inflammatory lymphoplasmacytic infiltration and collagen deposition in mesenteric adipose tissue without signs of obliterative phlebitis (Figure 2A).

When the immunohistochemical staining for IgG (Figure 2B) and IgG4 (Figure 2C) plasma cells was performed, an IgG4 positive cells/IgG positive cells ratio of 50% was found and an average of 20 IgG4 positive cells/high-power field (HPF) were counted in five different HPF.

The diagnosis of IgG4-RSM was made in one month and the patient was started on oral 0.6 mg/kg methylprednisolone, which was gradually tapered over six months and then stopped. The patient's one-year control of both abdomen CMCT and total body 18F-FDG PET/CT revealed no disease recurrence and the above-mentioned blood examinations reverted to normal range, namely IgG4 levels (85mg/dL). At the moment, the patient is still doing well.

## Discussion

Our case report describes a patient with symptomatic sclerosing mesenteritis as the only manifestation of IgG4-RD. The above-mentioned abdominal symptoms along with the results of both the abdomen CMCT and total body 18F-FDG PET/CT initially made us suspect the presence of a malignant tumor. However, the increase of serum IgG4 levels and the histopathological results of IgG4/IgG ratio of 50% with 20 IgG4 positive plasma cells/HPF, allowed us to make a definite diagnosis of IgG4-RSM in accordance with the commonly accepted 2011 comprehensive diagnostic criteria (CDC) of IgG4-RD, updated in 2017 [5,6]. The excellent response to corticosteroid therapy was in agreement with literature data from a consensus statement of 17 Japanese centers, according to which the prednisolone daily dose of 0.6 mg/kg is recommended as the first-line treatment for IgG4-RD [1].

Epidemiologic studies concerning IgG4-RD mainly come from Japan, where the incidence of this disease has been estimated to be 0.28-1.08/100,000 with 336-1,300 patients newly diagnosed per year [4]. Moreover, it is known that the majority of patients are men, generally older than the age of 50 [7,8]. IgG4-RSM is an even more rare entity characterized by focal or diffuse fibrosis and inflammatory changes in the small bowel mesentery that can appear as a soft tissue mass that may narrow or encase the adjacent blood vessels [9,10] and can also cause bowel obstruction [11]. Such fibrotic and inflammatory alterations account for the avid uptake of FDG on PET [12,13]. Main potential mimics include lymphoma and carcinoid or neuroendocrine tumors, which will also exhibit FDG avidity on PET imaging. Hence, its preoperative diagnosis remains challenging. In this regard, one useful finding is the preservation of the fat around the mesenteric vessels seen in IgG4-RSM [9].

The 2017 CDC for IgG4-RD largely reflects that of 2011. The definitive diagnosis of IgG4-RD is made in patients when the following three items are all positive: (1) characteristic diffuse/localized swelling or masses in single or multiple organs; (2) a serum IgG4 concentration >135 mg/dL; (3) histological findings of marked lymphocyte and plasmacyte infiltration and fibrosis as well as IgG4-positive plasma cell infiltration: ratio of IgG4/IgG positive cell >40%, and IgG4-positive plasma cells/HPF >10. A diagnosis of IgG4-RD is probable when patients fulfill criteria 1 and 3, whereas a probable diagnosis of IgG4-RD concerns patients who meet criteria 1 and 2. Patients with a possible or probable diagnosis could be re-diagnosed by organ-specific IgG4-RD criteria, e.g., for the pancreas, bile ducts, kidneys, lungs, and ocular cavity [6].

Keeping in mind the aforementioned CDC 2017, we undertook a complete MEDLINE, EMBASE, Web of Science, and Scopus database research of all case reports of IgG4-RSM published so far since 2003. Such criteria were strictly applied and, to the best of our knowledge, were met overall in five cases with definite diagnoses (Table 2).

**Table 2:** Summary of case reports of IgG4-RSM with 2017 CDC fulfilled for definite diagnosis. IgG4-RSM: IgG4-related sclerosing mesenteritis; CDC: comprehensive diagnostic criteria; HPF: high power field; IgG4-RD: immunoglobulin G4-related disease; Ref.: reference; M: male; F: female; PC: present case.

Case	Age (y)	Sex	Serum IgG4 (mg/dL)	Sample	Immunochemistry IgG4+/HPF	Immunochemistry IgG4+/IgG+ (%)	Fibrosis	Obliterative phlebitis	IgG4-RD in other sites	Ref.
1	82	F	171	Resection	130	75.9	Yes	Yes	None	[14]
2	51	M	189	Resection	<45	50	Yes	Yes	None	[15]
3	67	M	145	Resection	>50	>40	Yes	No	None	[16]
4	64	M	665	Resection	>100	55.4	Yes	No	None	[17]
5	38	M	457	Resection	70	64	Yes	No	None	[18]
6	73	F	145	Resection	20	50	Yes	No	None	PC

Notably, based on the CDC 2017 for IgG4-RD, the definite diagnosis was excluded in a number of case reports of sclerosing mesenteritis because the minimum IgG4 serum level of 135 mg/dL was not reached, and/or the requirements for the aforementioned histopathologic findings were not met.

With regard to treatment, the patient of case no. 3 [16] refused steroid therapy, while the remaining five cases were similarly treated with a starting dose of 0.6 mg/kg/day of methylprednisolone, gradually tapered over the course of six months, and remained asymptomatic off steroids one year after. We can therefore infer that steroid therapy is associated with a favorable prognosis.

## Conclusions

Our case report highlights the mesentery as an uncommon site of involvement among IgG4-RD and stresses the central role of both imaging and surgery as there are many malignant mimics in the differential diagnosis. The imaging demonstration of a diffuse mesenteric swelling, along with the increase of serum IgG4 levels and the mesenteric histopathological evidence of IgG4-RD, proved decisive for the patient’s outcome. As IgG4-RSM usually mimics the imaging characteristics of mesenteric malignancies, its preoperative diagnosis remains challenging. The following excellent response to corticosteroid therapy was in agreement with literature data according to which such therapy is recommended as the first-line treatment for IgG4-RD, showing a favourable prognosis in most cases. 

Finally, our extensive and in-depth research on previously reported cases showed that IgG4-RSM is indeed rare, as only six cases with a definite diagnosis have been reported so far. Our paper is part of ever-increasing literature on IgG4-RD because three of the six cases have been reported in the last two years. IgG4-RD recognition is increasing around the world, and clinicians from nearly every specialty are now becoming confident with most of its manifestations.
